# Prevalence and Risk Factors of Dyslipidemia among Type 2 Diabetes Patients at a Referral Hospital, North Eastern Ethiopia

**DOI:** 10.4314/ejhs.v31i6.23

**Published:** 2021-11

**Authors:** Worku Misganaw Kebede, Kefyalew Dagne Gizachew, Getaneh Baye Mulu

**Affiliations:** 1 Department of Nursing, College of Health Sciences, Debre Berhan University, Debre Berhan, Ethiopia; 2 Department of Psychiatry, College of Health Sciences, Debre Berhan University, Debre Berhan, Ethiopia

**Keywords:** Dyslipidemia, prevalence, risk factors, diabetes, Debre Berhan

## Abstract

**Background:**

The prevalence of diabetes and its associated complications rising in Ethiopia ranged from 2.0%–6.5%, the primary cause of morbidity and mortality with consequential economic impact. This study aimed to assess the prevalence and risk factors of dyslipidemia among diabetes follow-up patients.

**Methods:**

Institution-based cross-sectional study was conducted at Debre Berhan Referral Hospital from January to March 2020 in 347 Adult type 2 diabetes follow-up patients using a convenient sampling technique. Data were collected by interviews and entered using Epi-data 4.2 and analyzed using SPSS version 25. Factors having a p-value < 0.25 in the bi-variable logistic regression model were entered into a multivariable logistic regression model. Statistical Significance was declared at a p-value ≤ of 0.05.

**Results:**

The prevalence of dyslipidemia among type 2 diabetes patients in this study was 59 %. Significantly associated variables were being female [AOR 2.6 (95% CI 1.2–3.2), P = 0.011], smoking history [AOR 4.1 (95% CI 2–6.8), P = 0.001], Being overweight [AOR 3.5 (95% CI 1.6–7.8), P = 0.002], Being obese [AOR 4.8 (95% CI 1.7–13), P = 0.002].

**Conclusion:**

Prevalence of dyslipidemia was high among diabetic patients, which accounts for 59%. Being female, smoking history, being overweight, and being obese were determinants of dyslipidemia. Patients with poor glycemic control need additional lipid-lowering therapies to prevent secondary Atherosclerotic vascular complications.

## Introduction

Urbanization and lifestyle change in the 20-first century increased the prevalence of chronic non-communicable diseases. As a result, modifiable risk factors were the main risk factors of diabetes and its complications. Studies showed a significant association of coronary events with raised lipoprotein B, total cholesterol, LDL cholesterol, and non-HDL cholesterol, on the other hand, decrease in HDL cholesterol level. This implies that the rise in bad cholesterol and reducing good cholesterol will have many adverse effects (1). As a result, there are more than 500 million cases of type 2 diabetes worldwide, and its prevalence becoming comparably high in low-income countries by the end of 2030 (2).

Lipid metabolism disorder or dyslipidemia in people with diabetes is the main predisposing factor to cardiovascular risk in patients with type 2 diabetes. It includes abnormal quantitative, qualitative, and kinetic lipoproteins that are primarily atherogenic. characterized by hypertriglyceridemia, low HDL-C, and mildly elevated LDL-C with a high proportion of small dense LDL cholesterol (3, 4).

Dyslipidemia is higher in patients with type 2 diabetes and is the most common modifiable risk factor of cardiovascular diseases among these populations. A nationwide study in Thailand population that the prevalence of dyslipidemia was 88.9%. Atherosclerotic cardiovascular disease secondary to dyslipidemia contains coronary heart disease, cerebrovascular disease, or peripheral artery disease. Diabetes without risk factor for cardiovascular disease and treatment for dyslipidemia after 10-year the risk of CVD will increase ≥by 7.5%. Besides, higher LDL-C was correlated with a 13.5% increased chance of 10-year risk for CVD. The significant risk factors of morbidity and mortality in patients with diabetes impose substantial health care expenditure (6,7).

Communities in low-income countries had poor knowledge and adherence to antilipidemic drugs to effectively manage dyslipidemia in type 2 diabetes patients. Without strict adherence to treatment modalities and lifestyle modification, patients could not reach the goal of the therapy. Atherogenic dyslipidemia was the leading risk factor to increase the risk of silent myocardial infarction and other coronary artery and coronary heart disease among type 2 diabetes patients, most commonly with elevated serum low-density lipoprotein (9, 10).

Ethiopia is one of the rapidly growing countries where urbanization, lifestyle change, and socio-economically developing. The prevalence of diabetes and its associated complications rising in Ethiopia ranged from 2.0%–6.5%, the primary cause of morbidity and mortality with consequential economic impact. A comparative study conducted in Tukur Anbessa specialized hospital, Addis Ababa, Ethiopia, revealed that those with poor glycemic control had elevated serum cholesterol levels than those with good glycemic control (11). (12).

Besides, cardiovascular risk and poor glycemic control dyslipidemia have a higher economic burden. Medications for lipid-lowering are costly, and it incurs higher expenditures annually. Generally, health care expenditures among patients with a cardiovascular risk had higher expenses over time. The costs were for emergency visits, outpatient visits, and pharmacy prescriptions (13).

Nowadays, cardiovascular risk factors are being prevalent in the general population. In addition, modifiable risk factors like hypertension, diabetes, dyslipidemia, obesity, smoking, abdominal obesity are persistently rising. As a result, cardiovascular risk also increases in the same fashion (14).

A general survey in India showed that nearly one-fourth (27%) of the participants have dyslipidemia from the general population. In addition, they have either triglycerides or cholesterol. Nine-point eight percent had hypercholesterolemia and 21.6% hypertriglyceridemia in the general population, both in urban and rural areas (15).

Diabetes is increasing promptly in the least developed states like Ethiopia (16). Comparably, dyslipidemia will increase even if studies did not show the exact magnitude, particularly in the study area. Therefore, this study aimed to assess the prevalence of dyslipidemia and its risk factors among type 2 diabetes follow-up patients attending Debre Berhan Referral Hospital.

## Materials and Methods

**Population and study design**: This study was conducted at the Debre Berhan Referral Hospital in diabetes follow-up clinic, Debre Berhan, Central Ethiopia. Adult diabetes patients who volunteered to give informed written consent were involved. An institution-based cross-sectional study was carried out from January to March 2020. Those study subjects who were pregnant were excluded from the study.

**Sample size determination and sampling approach**: The sample size was determined using a single population proportion formula by considering 65.6% dyslipidemia prevalence in Kembata Tembaro zone southwest Ethiopia (4) and assumptions of 5% sampling error, 10% for those potentially eligible participants who will not respond, and 95% level confidence level the final sample size was 347.

While Outcome variable was dyslipidemia, Predictor variables were **Social and demographic elements** (Age of participants, sex of participants, religion, level of education, and marital status of participants), **Behavioral and Clinical characteristics** (smoking, alcohol drinking, Blood pressure, reference diastolic blood pressure, reference systolic blood pressure, types of medications, duration on medications, types of medications, and dyslipidemia) and **Biochemical characteristics (**Total cholesterol, Triglycerides, Low-Density Lipoprotein (LDL), High-Density Lipoprotein (HDL), HgbA1c).

**Sampling technique, procedure, and data collection tools**: Data were collected by six nurses working on diabetic follow-up clinics after the participants agreed to written informed consent. A convenient sampling technique was used to select study subjects from the study population; this sampling technique was used because it was difficult to use a random sampling technique as the study subjects' appointment for their follow-up varied, and some of them might not come for the follow-up on the specified date. In addition to this, it is easy and not time-consuming compared to other sampling techniques. Written consent was obtained from each study subject before any data collection. An interview-administered structured questionnaire was used to collect sociodemographic and clinical data.

Anthropometric characteristics were taken by trained professional nurses working at the diabetic clinic in the morning after overnight fasting by using a standardized protocol. First, the height and weight of each study subject were measured by using an analog-digital scale without shoes. The height was measured by instructing each subject's feet pointed outward; legs straight and knee together; arms at sides; head, shoulder blades, buttocks, and heels touching measurement surface; looking straight ahead, and shoulder relaxed. Next, the body mass index (BMI) was calculated using the formula, weight over height square, and recorded results.

Circumferences were evaluated by using a stretch-resistant 1-cm-wide measuring tape that provides a constant measurement. Circumference measurements were taken while the subject is in the standing position and breathing normally. The tape was snug around the body for taking waist circumference measurements but not pulled so tight that it is constricting. Each measurement was repeated twice; the average of measurements within 1 cm of one another was calculated. If the difference between the two measurements exceeds 1 cm, the two measurements were repeated. BP was measured by using a mercury sphygmomanometer two consecutive times. The first measurement was taken after a person rest for at least 15 minutes before taking a meal, medication, and caffeine. Then the first measurements were taken in the early morning, and the second measurements were repeated after 6 hrs. After that, the BP values used for analysis were the mean of the two measurements.

Blood specimen collection technique and investigation Five milliliters of blood were collected from each study subject by a trained medical laboratory technologist after overnight fasting following the standard operating procedure guideline. A blood sample was not collected for clients with laboratory results not more than one month in their follow-up uptime. The analysis was performed using the A25 Biosystems clinical chemistry analyzer (Biosystems, Costa Brava, Spain) at Debre Berhan Referral Hospital Laboratory Unit.

**Operational definitions**: Hypertension was defined as systolic BP (SBP; ≥140 millimeters of mercury [mmHg]) or diastolic BP (DBP; ≥90 mmHg); both SBP and DBP are elevated in patients on antihypertensive medication (17).

Considering their current fasting blood glucose (FBG) level, participants were classified as with good glycemic control (FBG <150 mg/dl) and poor glycemic control (FBG ≥150 mg/dl) (18). Dyslipidemia was defined as a lipid profile that consists of the following abnormalities either singly or in combination. These include TC ≥200 mg/dL, TG levels ≥150 mg/dL, HDLC <40 mg/dL, and LDL-C ≥100 mg/dL. Based on the results of BMI, study subjects were categorized as underweight with BMI <18.5 kg/m^2^; normal weight when BMI range was 18.5 to 24.9 kg/m^2^; overweight with BMI range from 25 to 29.9 kg/m2; obese with BMI range from 30 to 34.9 kg/m^2^; severely obese with BMI range from 35 to 39.9 kg/m^2^; and morbidly obese with BMI range ≥40 kg/m^2^.

**Data quality assurance**: To assure the quality of the data, one-day training was given for data collectors and supervisors before data collection. A pre-test was conducted on 5% of the sample size in Ayu General Hospital before the actual data collection process. The questionnaire was carefully designed, and the English version was used for data collection. Before the actual data collection time, the questionnaire was checked for clarity and completeness. The supervisor has monitored the data collection process by checking the completeness and correcting the data collection site. The principal investigator has checked the data for its completeness during data entry and the cleaning process.

**Data processing and analysis**: The collected data were checked for completeness, coded, and then entered into Epi-Data Version 4.2.1 then exported to SPSS version 24 for analysis. Descriptive statistics were used to describe the study participants about relevant variables. To determine the actual predictors of chronic kidney disease, binary logistic regressions were applied. The variables found to have a p-value of <0.2 with the outcome variable at bivariable analysis were entered into a multivariable logistic regression model. Moreover, the variables with significant associations were identified based on p-values < 0.05 and AOR (adjusted odds ratio), with 95% CI to measure the strength of the associations. Finally, the results of the study were presented through tables, figures, and text.

**Ethical Approval:** Ethical clearance was obtained from the Debre Berhan University Research Ethics and Publication Committee before conducting the study. Further permission was obtained from Debre Berhan Referral Hospital administration. Any information related to the personal identification of the study participants was not recorded to maintain the confidentiality of the study. Written consent was obtained from all caregivers.

## Results

**Socio-demographic characteristics**: Three hundred twenty-seven diabetic patients were fulfilling's inclusion criteria, given a response rate of 94.2 %. The mean age of the participants was 53 + 17 years, and their mean diabetic follow-up period was eight years. The mean BMI of the participants was 26 + 3.8 kg/m^2^. The mean waist circumference was 81.9 + 8.8 cm. above half of the study subjects were females, and over two-thirds of the respondents lived in urban areas (Table 1).

**Table 1:** Socio-demographic and behavioral characteristics of Type 2 diabetes follow-up patients at Debre Berhan Referral Hospital (n = 327)

Variables	Characteristics	Dyslipidemia			
		Yes		No	
		
		N	%	N	%
**Age**	<60	137	71	106	79.1
	>60	56	29	28	20.9
**Sex**	Male	97	50.3	80	59.7
	Female	96	49.7	54	40.3
**Residence**	Urban	73	37.8	39	19.1
	Rural	120	62.2	95	70.9
**Family Hx of Dm**	Yes	51	26.4	47	35.1
	No	142	73.6	87	64.9
**Smoking**	Yes	64	33.2	19	14.2
	No	129	66.8	115	85.8
**Alcoholic**	Yes	63	32.6	33	24.6
	No	130	67.4	101	75.4

DM: diabetes mellitus, HX: history

**Clinical and biochemical characteristics**: The clinical characteristics of the participants showed that 257 (78.6%) of them were in the follow-up duration of below ten years of age. Regarding their blood pressure measurement, nearly half of the study subjects, systolic blood pressure was below 130 mmHg. Furthermore, their lipid profile analysis showed that three-fourth of the study participant's HDL level was less than 60g/dl. Besides this, they showed that nearly two-thirds of the study participant's total cholesterol level was less than 200 g/dl (Table 2).

**Table 2:** Showing clinical and biochemical characteristics of Type 2 diabetes follow-up patients at Debre Berhan Referral Hospital (n = 327)

				Dyslipidemia			
Variables	Characteristics	Freq.	%	Yes	%	No	%
Serum HbAlc	<7	98	30	53	27.5	45	33.6
	>7	229	70	140	72.5	89	66.4
Systolic BP	<130	175	53.5	99	51.3	76	56.7
	>130	152	46.5	94	48.7	58	43.3
Diastolic BP	<80	211	64.5	119	61.7	92	67
	>80	116	35.5	74	38.3	42	33
Serum Creatinine	<1.2 mg/dl	295	90.2	161	83.4	122	91
	>1.2 mg/dl	32	9.8	32	16.6	12	9
Total cholesterol	<200 mg/dl	209	63.9	99	51.3	110	82.1
	>200 mg/dl	118	36.1	94	48.7	24	17.9
Triglyceride	<100 mg/dl	189	57.8	92	47.7	97	72.4
	>100mg/dl	138	42.2	101	52.3	37	27.6
HDL	<60 mg/dl	274	83.8	160	82.9	114	85.1
	>60 mg/dl	53	16.2	33	17.1	20	14.9
	Normal weight	85	26	43	22.3	42	21.8
	overweight	183	56	113	58.5	70	52.2
BMI/kg/m^2^	Obese	59	18	37	19.2	22	26
	<78.75	123	37.6	76	39.4	47	35.1
	78.75–87.5	93	28.4	57	29.5	36	26.9
Waist circumference/cm	>87.5	111	34	60	31.1	51	38

BP: Blood Pressure, HbA1c: Glycated Hemoglobin, HDL: High-Density Lipoprotein, LDL: Low-Density Lipoprotein, BMI: body mass index, cm: centimeter

**Prevalence of Dyslipidemia**: Prevalence of dyslipidemia was assessed; the higher prevalence of isolated dyslipidemia in this study was low HDL 274(83.8%). In addition, combined cholesterol level showed that HDL(low) and high LDL was 160(48.9%) (Table 3).

**Table 3:** Prevalence of lipid abnormality among type 2 diabetes follow-up patients

Lipid abnormality		N (%)
Isolated	High total cholesterol	118(36.1)
	High triglyceride (TG)	138(42.2)
	HDL (low)	274(83.8)
	High LDL	193(59)
Combined	HDL (low) and High LDL	160(48.9)
	HDL (low) and TG	120(36.7)
	High LDL and TG	101(30.9)

HDL, high-density lipoprotein; LDL, low-density lipoprotein; TC, total cholesterol; TG, triglyceride

**Associated factors of dyslipidemia**: Those Factors like age, sex, residence, family history, smoking history, glycated hemoglobin, duration of diabetes, diastolic BP, BMI, and waist circumference had a P-value of less than 0.25 in the bi-variable logistic regression model. Therefore, those variables were entered into multivariable logistic regression for further analysis. Variables having a statistically significant association in multivariable logistic regression were sex, smoking history, and BMI.

Being female was 2.6 times more likely to develop dyslipidemia than their counterparts [AOR 2.6 (95% CI 1.2–3.2), P = 0.011]. Likewise, participants with smoking history were four times the odds of developing dyslipidaemia than non-smokers [AOR 4.1 (95% CI 2–6.8), P = 0.001]. Furthermore, being overweight was 3.5 times more likely to develop dyslipidaemia than those with normal weight [AOR 3.5 (95% CI 1.6–7.8), P = 0.002]. Also, being obese was 4.8 times more likely to develop dyslipidemia than those who have normal weight [AOR 4.8 (95% CI 1.7–13), P = 0.002] (Table 4).

**Table 4:** Bivariable and multivariable logistic regression analysis of dyslipidemia among type 2 diabetes follow-up patients at Debre Berhan Referral Hospital (n = 327)

Variables	Category	Dyslipidemia					
						COR	AOR 95 % CI
		Yes =193		No =134			
		N	%	N	%		
**Sex**	Male	97	50.3	80	59.7	1	
	Female	96	49.7	54	40.3	1.5(0.9–2.3)	2.6(1.2–3.2) *
**Age**	<60	137	71	106	79.1	1	1
	>60	56	29	28	20.9	1.5(0.9–2.6)	1.7(0.9–2.9)
**Smoking history**	Yes	64	33.2	19	14.2	3(1.7–5.3)	4.1(2–6.8) ***
	No	129	66.8	115	85.8	1	1
**Alcoholic history**	Yes	63	32.6	33	24.6	1.5(0.9–2.4)	1.1(0.6–2)
	No	130	67.4	101	75.4	1	1
**Diastolic BP**	<80mmHg	119	61.7	92	67	1	1
	≥80mmHg	74	38.3	42	33	1.5(0.9–2.2)	1.1(0.6–1.7)
**HbA1c**	<7%	53	27.5	45	33.6	1	1
	≥7%	140	72.5	89	66.4	1.3(0.8–1.9)	1.3(0.8–2.3)
**BMI kg/m^2^**	Normal weight	43	22.3	42	21.8	1	
	Overweight	113	58.5	70	52.2	1.6(0.9–2.7)	3.5(1.6–7.9) **
	Obese	37	19.2	22	26	1.6(0.8–3.2)	4.7(1.7–13) **
**Waist **	<78.75	76	39.4	47	35.1	1	
**circumference/cm**	78.75–87.5	57	29.5	36	26.9	0.9(0.7–1.7)	0.5(0.2–1.1)
	>87.5	60	31.1	51	38	0.7(0.4–1.2)	0.3(0.1–0.6)
**Residence**	Urban	73	37.8	39	19.1	1.5(0.9–2.4)	1.6(0.9–2.7)
	Rural	120	62.2	95	70.9	1	

**Note:**

* Statistically significant with P-value ≤0.05

** statistically significant with P-value ≤ 0.01

*** Statistically significant with a P-value ≤ 0.001

AOR: adjusted odds ratio, BP: blood pressure CKD: chronic kidney disease, COR: crude odds ratio, HDL: high-density lipoprotein, LDL: low-density lipoprotein

## Discussion

In this study, the prevalence of dyslipidemia among type 2 diabetes patients was relatively high. In addition, determinants among adult type 2 diabetes follow-up patients at Debre Berhan Referral Hospital were identified.

Despite 40% and 21.8% of the study participants were taking atorvastatin and simvastatin, respectively, the prevalence of dyslipidemia among type 2 diabetes patients was high, 193 (59%). All of the participants were type 2 DM patients. In isolation prevalence of dyslipidemia were: 118 (36.1%) High total cholesterol, 138 (42.2%) High triglyceride (TG), and 274 (83.8%) HDL (low).

When comparing our results to those of previous studies, in this study, the prevalence of dyslipidemia was lower than across sectional studies conducted in Durame southern nation nationality Ethiopia (4), in Johannesburg South Africa (19) in west Nigeria (20), and Thailand (6). However, this difference in dyslipidemia prevalence might be because of variation in the characteristics of the study subjects.

However, this finding is higher than studies done in Saudi Arabia (21) in China (22), India (23). This might be due to time differences and socio-demographic characteristics, and these studies were carried out among the healthy rural population.

In this study, Sex differences were a significant association with dyslipidemia. Being female had a significant association with dyslipidaemia than their counterparts [AOR 2.6 (95% CI 1.2–3.2), P = 0.011]. This is consistent with what has been found in previous studies done in Durame general hospital southern nation nationality (4), in Jordan (24), in Iran (25), in Turkey (26), and adults Chinese population (27).

According to this study, smoking history was a significant risk factor for dyslipidemia. DM patients with smoking history were four times more likely to develop dyslipidemia than those who didn't have a smoking history [AOR 4.1 (95% CI 2–6.8), P = 0.000]. This was in agreement with other related studies done in Taiwanese (28), and in India, it was revealed that serum cholesterol level was significantly higher in chronic smokers than non-smokers. (29). This might be due to the difference in socio-economic and lifestyle.

Furthermore, in this study, we also found that BMI was a significant risk factor for dyslipidemia. Patients who were overweight and obese were significantly associated with dyslipidemia than those having a normal weight. Being an overweight and obese were 3.5 and 4.8 times respectively more likely to develop dyslipidaemia than those who have normal weight [AOR 3.5 (95% CI 1.6–7.8), P = 0.002], [AOR 4.8 (95% CI 1.7–13), P = 0.002]. These findings are similar to those reported by studies conducted in Durame general hospital's southern nation nationality, Ethiopia (4), in Saudi Arabia (21).

In conclusion, the finding of this study showed that there is a higher prevalence of dyslipidemia among type 2 diabetes patients, which accounts for 59%. Being female, smoking history, and having high BP were the associated risk factors. Patients with poor glycemic control need additional lipid-lowering therapies to prevent secondary Atherosclerotic vascular complications. The limitation of the study was the issue of representativeness due to the convenient sampling technique.
